# Real-time vision, tactile cues, and visual form agnosia: removing haptic feedback from a “natural” grasping task induces pantomime-like grasps

**DOI:** 10.3389/fnhum.2015.00216

**Published:** 2015-05-06

**Authors:** Robert L. Whitwell, Tzvi Ganel, Caitlin M. Byrne, Melvyn A. Goodale

**Affiliations:** ^1^Graduate Program in Neuroscience, The University of Western OntarioLondon, ON, Canada; ^2^Department of Psychology, The University of Western OntarioLondon, ON, Canada; ^3^The Brain and Mind Institute, The University of Western OntarioLondon, ON, Canada; ^4^Department of Psychology, Ben-Gurion University of the NegevBeer-Sheva, Israel; ^5^Department of Physiology and Pharmacology, The University of Western OntarioLondon, ON, Canada

**Keywords:** grasping, pantomime grasps, haptic feedback, visual feedback, visual form agnosia

## Abstract

Investigators study the kinematics of grasping movements (prehension) under a variety of conditions to probe visuomotor function in normal and brain-damaged individuals. “Natural” prehensile acts are directed at the goal object and are executed using real-time vision. Typically, they also entail the use of tactile, proprioceptive, and kinesthetic sources of haptic feedback about the object (“haptics-based object information”) once contact with the object has been made. Natural and simulated (pantomimed) forms of prehension are thought to recruit different cortical structures: patient DF, who has visual form agnosia following bilateral damage to her temporal-occipital cortex, loses her ability to scale her grasp aperture to the size of targets (“grip scaling”) when her prehensile movements are based on a memory of a target previewed 2 s before the cue to respond or when her grasps are directed towards a visible virtual target but she is denied haptics-based information about the target. In the first of two experiments, we show that when DF performs real-time pantomimed grasps towards a 7.5 cm displaced imagined copy of a visible object such that her fingers make contact with the surface of the table, her grip scaling is in fact quite normal. This finding suggests that real-time vision and terminal tactile feedback are sufficient to preserve DF’s grip scaling slopes. In the second experiment, we examined an “unnatural” grasping task variant in which a tangible target (along with any proxy such as the surface of the table) is denied (i.e., no terminal tactile feedback). To do this, we used a mirror-apparatus to present virtual targets with and without a spatially coincident copy for the participants to grasp. We compared the grasp kinematics from trials with and without terminal tactile feedback to a real-time-pantomimed grasping task (one without tactile feedback) in which participants visualized a copy of the visible target as instructed in our laboratory in the past. Compared to natural grasps, removing tactile feedback increased RT, slowed the velocity of the reach, reduced in-flight grip aperture, increased the slopes relating grip aperture to target width, and reduced the final grip aperture (FGA). All of these effects were also observed in the real time-pantomime grasping task. These effects seem to be independent of those that arise from using the mirror in general as we also compared grasps directed towards virtual targets to those directed at real ones viewed directly through a pane of glass. These comparisons showed that the grasps directed at virtual targets increased grip aperture, slowed the velocity of the reach, and reduced the slopes relating grip aperture to the widths of the target. Thus, using the mirror has real consequences on grasp kinematics, reflecting the importance of task-relevant sources of online visual information for the programming and updating of natural prehensile movements. Taken together, these results provide compelling support for the view that removing terminal tactile feedback, even when the grasps are target-directed, induces a switch from real-time visual control towards one that depends more on visual perception and cognitive supervision. Providing terminal tactile feedback and real-time visual information can evidently keep the dorsal visuomotor system operating normally for prehensile acts.

## Introduction

Being able to reach out and grasp objects with considerable skill is one of the defining features of primates. The act itself typically involves the use of real-time visual information and is directed at a visible object. It also results in contact with the object, manipulation, and haptic feedback. Detailed analysis of movements of the fingers, hand, and wrist show that the posture and orientation of the moving hand reflect the geometric properties of the goal object (e.g., Jeannerod, 1988; Jakobson and Goodale, 1991; Paulignan et al., 1991a,b; Gentilucci et al., 1996). The visually-mediated control of grasping is thought to involve the dorsal stream of visuomotor pathways in the posterior parietal cortex (PPC) and their interconnections with premotor areas of the frontal lobe (for review see: Culham and Valyear, 2006; Grafton, 2010; Davare et al., 2011). In line with this view, disrupting the activity of the anterior areas of the intraparietal sulcus of the PPC with transcranial magnetic stimulation (TMS) affects the grasp kinematics in neurologically healthy individuals (e.g., Glover et al., 2005; Tunik et al., 2005; Rice et al., 2006, 2007). Furthermore, damage to dorsal-stream structures in the PPC can result in selective visuomotor deficits involving misreaching and/or poor grasp formation (Jeannerod, 1986; Perenin and Vighetto, 1988; Jakobson et al., 1991; Goodale et al., 1994a; Jeannerod et al., 1994; Binkofski et al., 1998; Milner et al., 2001; Karnath and Perenin, 2005; Cavina-Pratesi et al., 2010). Despite their deficits in real-time visuomotor control, however, some patients with dorsal-stream lesions show relatively preserved visual perceptual abilities on comparable tasks that require object form processing (Jakobson et al., 1991; Goodale et al., 1994a; Jeannerod et al., 1994; Milner et al., 2001).

In contrast to the effects of lesions to the dorsal stream, lesions that are largely restricted to the ventral stream often produce gross deficits in the ability to report the features of visual stimuli, such as color, visual texture, and form. A deficit in form vision is typically referred to as “visual form agnosia” (for review, see Goodale and Milner, 2013). One of the best known examples of such a patient is DF (Milner et al., 1991; for review see Whitwell et al., 2014b; but see also patients JS and MC; Wolf et al., 2008; Karnath et al., 2009, respectively). DF and other similar patients had sustained bilateral lesions of varying extent to occipito-temporal cortex and, as a result, were left with a persistent deficit in visual form perception. Nevertheless, when these patients reached out and grasped objects, that they failed to discriminate amongst, the online configuration of their grasping hand reflected the spatial and geometric properties of those objects (Goodale et al., 1991, 1994a,b; Marotta et al., 1997; Westwood et al., 2002; Wolf et al., 2008; Karnath et al., 2009; Whitwell et al., 2014a, in press). Their relatively normal performance is made all the more remarkable by the fact that these patients were all demonstrably at chance when asked to manually indicate the widths of exemplars from a set of so-called “Efron blocks” (Efron, 1969) placed directly in front of them. The Efron blocks vary in length and width but, critically, are matched for cues that these patients, including DF, can perceive such as weight, texture, color, and overall surface area. In other words, despite gross deficits in visual object perception, these patients were capable of making relatively normal-looking visually guided target-directed actions, such as reaching and grasping, presumably by virtue of having spared visuomotor networks in the dorsal stream. These studies, together with the complementary neuropsychological studies of patients with dorsal-stream lesions described above, as well as demonstrations of dissociations between perceptual report and visually guided actions in normally-sighted individuals, (e.g., Ganel et al., 2008; Stöttinger et al., 2010, 2012) have provided compelling support for the Two Visual Systems Hypothesis (TVSH; Goodale and Milner, 1992; Milner and Goodale, 2006), which in turn has influenced subsequent and expanded proposals on the functional organization of the primate visual system (Rizzolatti and Matelli, 2003; Kravitz et al., 2011, 2013).

In a seminal investigation, Goodale et al. (1994b) explored the dependence of the dorsal stream on real-time visual control by examining how normal DF’s grasps looked when she was forced to rely on a memory of a recently previewed target. To do this, the authors compared natural grasps to a variant Milner et al. (2001) later-called “delayed-pantomimed grasping” (DPG) in which the participants, including DF, executed grasps to the remembered location of targets viewed as recently as 2 s before the cue to respond occurred. In this task, the participants’ view of the workspace was restored following the delay period. Critically, however, the experimenter removed the object during the delay period and so it was no longer present when the participants were cued to reach out and pretend to pick up the remembered object “as if it was still physically present” (p. 1165). The DPG task therefore differed from the natural grasping task in two respects: (1) online visual input about the target was not available when the response was cued; and (2) no haptics-based object information was available at the end of the movement. The results showed that all of the participants, including DF, moved their hand towards the previewed location of the target. Nevertheless, there were some clear differences in the hand kinematics of the two grasping tasks. Compared to natural grasps, the DPGs of the participants, including DF, took longer to complete, exhibited slower peak hand velocities, and showed smaller anticipatory grip aperture. The measure on which DF’s performance differed most-drastically from that of the controls was the in-flight, anticipatory adjustments in grip aperture to the widths of the remembered targets (grip scaling). Whereas the controls showed no change in their grip scaling slope (relating grip aperture to target width) moving from natural grasps to DPGs, DF’s slope bore no relationship whatsoever to target width. Goodale et al. argued that DF’s failure in the DPG task was due to her inability to form a visual percept of the target and extract its width. Their reasoning was based on two assumptions: (1) that the DPG task required participants to use a remembered percept of the target’s width; and (2) that the creation of this percept required an intact object processor housed in the occipital-temporal cortex. Thus, their argument runs, DF’s failure in grip scaling was a direct result of the damage to her ventral stream, preventing her from forming a visual percept in the first place to store in memory.

Importantly, Goodale et al. (1994b) also tested DF and the controls in an additional variant of the “natural” grasping task. In this new task, the participants, including DF, were presented with a visible Efron block and were asked to imagine an identical version of that object displaced to the right of it (7.5 cm), and then to reach out to grasp this imagined object “as if it were physically present” (Goodale et al. p. 1171–1172). Unlike the DPG task, this real-time displaced-pantomime grasping (RPG) task allowed the participants a full view of the workspace throughout the trial which included the Efron block and the hand and limb. Thus, the availability of real-time visual input about the object was equivalent across the natural and the RPG tasks, even though the target-directedness of the two tasks along with the availability of haptics-based object information clearly differed. Nevertheless, the results showed that, compared to natural grasps, the RPGs took longer to complete, exhibited slower peak hand velocities, and showed smaller anticipatory grip apertures. Thus, regardless of whether the pantomime grasps of neurologically-intact individuals are planned using online or remembered visual information about the object, removing haptics-based object information slows the hand movement, increases the movement time, and reduces the overall grip aperture. Noting an increase in the variability in DF’s anticipatory grip aperture for the RPG task, Goodale et al. ultimately concluded that both the DPG and RPG tasks produced catastrophic results for her grip scaling. Interestingly, however, in stark contrast to an absence of grip scaling in DF’s DPGs, DF’s grip aperture in the RPG task actually appears to be linearly related to the width of the target.

Common to both of Goodale et al.’s (1994b) pantomime tasks is an obvious requirement to pretend to pick up either the remembered or imagined target as if it were actually there and an absence of haptics-based object information. As we have already pointed out, the availability of real-time visual input following the cue to perform the grasp differed between the two tasks. Thus, this factor alone can reasonably account for any differences in DF’s performance across the two pantomime grasping task variants. As such, DF’s poor performance on the DPG task serves as a striking example of the dependence of some visuomotor tasks (pantomime grasps) on ventral stream processing, not only in DF but, presumably, in neurologically-intact individuals as well. One perhaps less obvious requirement of Goodale et al.’s tasks is the fact that the dimensions of the Efron blocks (only 1 cm in height) that were used in these experiments allowed the participants to receive tactile feedback from the surface of the table at the end of their reach. This was because the participants could not reasonably be expected to refrain from touching the surface of the table with their fingertips when simulating reaching out to pick up short rectangular blocks. Thus, the table may well act as a proxy when the grasps are directed next to the visible object. Importantly, haptics-based object information need not correlate with the visual size of targets for DF’s grip scaling to be normal. Indeed, when the grasped object remains an intermediate size despite changes in the visual size from trial to trial, DF’s grip aperture scales to the visual size (Whitwell et al., 2014a, in press). According to this view (see also Milner et al., 2012), both terminal tactile feedback *and* real-time visual input are critical for normal, dorsally-mediated prehension. Unfortunately, Goodale et al., did not compare DF’s performance in the RPG task directly against the performance of the controls, presumably because there were differences between DF and the controls in terms of the stimulus set (six Efron blocks vs. three) and the presentation protocol (one target position vs. three). Determining whether DF’s grip scaling in this task is in fact normal or abnormal would help rule out (or rule in) the importance of terminal tactile feedback for normal, real-time prehension. Therefore, in the first of our two experiments, we aimed to fill in this gap by revisiting DF’s grip scaling in Goodale et al.’s RPG task. We tested a new group of control participants using the same stimulus set and protocol that was used by Goodale et al. to determine whether DF’s real time-pantomime grasps were indeed as good as the controls and, more importantly, whether or not her grip scaling in this task would actually dissociate from that of her “natural” grasps as is commonly assumed.

## Experiment 1

### Methods

#### Participants

Eight self-reported right-hand dominant age-appropriate and gender-matched control participants ranging from 31 to 46 years of age (*M* = 39.1, SD = 5.7), volunteered to take part in the experiment to compare DF’s grip scaling in the natural grasping and RPG tasks. The controls provided written informed consent and were compensated $20 for their time. All experiments were approved by the local ethics committee and were in accordance with the Declaration of Helsinki.

#### Apparatus and Stimuli

**Figure 1:**
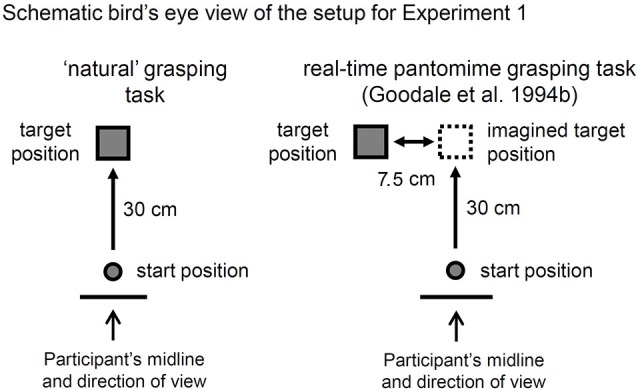
**A bird’s eye view of the setup for Experiment 1**. As outlined in Goodale et al. (1994b), the targets were six Efron blocks (varied widths and lengths but a constant surface area, weight, height, color and texture) positioned 30 cm from the start button along the participant’s midline in the “natural” grasping task. In the “real-time” pantomimed grasping (RPG) task, the target was positioned 7.5 cm to the right of the position used for the natural grasping task. DF (in Goodale et al.’s study) and the control participants (in the current study) were asked to imagine that the target was out in front of them, immediately to the right of its visible position and to reach out to grasp that imaginary target as if it were actually there. Notably, terminal tactile feedback was available when the hand made contact with the surface of the table at the end of the reach.

Details of the apparatus and stimuli used to test the controls for patient DF’s data set can be found in Goodale et al. (1994b). Briefly, the stimuli consisted of a set of Efron blocks that were 1 cm in height but varied in their lengths and widths as follows: *l* × *w (in cm)*, 10 × 2.5, 8.3 × 3, 7.1 × 3.5, 6.3 × 4, 5.6 × 4.5, 5 × 5. The kinematic data was collected at 200 Hz using an optoelectronic recording system (OPTO*TRAK* 3020, Northern Digital, Waterloo, ON, Canada) that recorded the 3D spatial locations of three infrared emitting diodes (IREDs). The IREDs were attached with adhesive tape at three positions on the right (grasping) hand: the distal left corner of the nail of the index-finger, the distal right corner of the nail on the thumb, and the skin blanketing the metacarpophalangeal joint (MCP) of the index-finger. The experimenter ensured that the pads of skin on the tip of the thumb and index-finger were uncovered to ensure normal tactile feedback from the goal objects when grasped. The leads from the IREDs were taped to the right forearm to ensure complete freedom of movement. There was only one target position, 30 cm along a sagittal plane from the start position. The start position was a raised button located 5 cm from the edge of the table facing the participant (see Figure 1). Before the experiment began, the experimenter ensured that all of the participants were seated comfortably and positioned close enough to the table so that they could grasp the objects at the farthest position comfortably and without leaning forward.

#### Procedure and Design

Details of the procedure and design used to test the controls can be found in Goodale et al. (1994b). Briefly, before each trial was initiated, the participants closed their eyes and held the tips of their right index-finger and thumb together while depressing the start button. The experimenter then gave a verbal prompt to the participant to open her eyes. The experimenter then waited approximately 2 s before giving a “go” signal for the participant to execute their response. For the natural grasping task, the participants were instructed to reach out, grasp across the width (near-far axis) of the Efron block, lift up, and put back down the Efron block using a precision grip (index-finger and thumb) as soon as they heard the go signal. At the beginning of the experiment, participants were asked to grasp the objects naturally: neither labored nor speeded. For the RPG task, the participants were instructed to imagine that the visible target to their left was positioned at the same distance along their midline (see Figure 1). They were further instructed to reach out to pick up the imaginary target as if it were physically there. The experimenter explained the procedure for the upcoming task before each block of trials. The experiment was comprised of 2 blocks of 36 trials each for a total of 72 trials. Each block of trials was dedicated to a different task. The block of natural grasps were performed before the block of RPGs. As Goodale et al. (1994b) cautioned, this order was chosen to give DF the maximum likelihood of being able to use the experience of actually grasping the objects when performing the RPGs. The order of the blocks were the same across all of the participants, including DF. For each block of trials (i.e., for each task), each one of the six Efron blocks was presented 6 times in a pseudorandom order.

#### Data Processing and Statistical Analysis

The data from the control participants were processed offline with custom software written in Matlab (Mathworks Inc., Natick, MA, USA). The positional information from the IREDs was low-pass filtered at 20 Hz using a 2nd order Butterworth digital filter. Grip aperture was computed as the Euclidean distance between the IRED placed on the thumb and the IRED placed on the index-finger, and the instantaneous velocities were computed for each of the three IREDs and for grip aperture. We analyzed three principal measures: peak grip aperture (PGA), the slope relating PGA to the target size, and the peak hand velocity (PHV). The PGA was defined as the largest grip aperture within a search window that was designed to capture the forward-reach component of the movement. The beginning of this window (the “movement onset”) was operationally defined as the first of 30 consecutive sample frames (150 ms) in which the velocity of the MCP IRED exceeded a threshold of 50 mm/s. Normally, one could use the movement onset as a measure of reaction time. In this case, however, because the timing between the initiation of the data collection and the subsequent experimenter’s verbal “go” command was free to vary (as was the case in Goodale et al., 1994b), reaction time (RT) could not be referenced to a fixed point in time. Thus, RT could not be computed reliably. Nevertheless, the end of the search window was defined as the first sample frame in which the velocity of the IRED fell below 75 mm/s. Linear regression of PGA on the widths of the Efron blocks was performed separately for each task and the resultant regression coefficient (i.e., the slope, *b*) relating the average increase in PGA (in mm) per incremental increase in Efron width (also in mm) was computed for DF and for the controls. The PHV was defined as the peak speed at which the MCP IRED travelled towards the target within the search window outlined above.

Notably, only DF’s PGA was available from the data set reported by Goodale et al. (1994b). Thus, only PGA and the slopes relating PGA to target size could be compared against the control data set. The PHV of the control participants was analyzed to test Goodale et al.’s finding that the RPGs of the controls are executed more slowly than natural ones in this slightly modified version of that task (one target position and six target sizes). The comparisons of interest in the control data were the differences in the PHV, overall PGA, and grip scaling slopes between the natural grasps and the RPGs. The comparisons of interest that involved DF included those measures that were common to both DF and the controls: the difference in overall PGA between the natural grasps and RPGs and the grip scaling slopes. A comparison of the PGAs between DF and the controls for each of the natural grasping and RPG tasks was not carried out given that inter-individual differences in IRED positioning and hand anatomy could have yielded spurious results. Comparisons of intra-individual differences involving PGA should be far less susceptible to this influence (if at all). Accordingly, we used independent-samples *t*-tests to assess the normality/abnormality of (1) DF’s slope on each of the two grasping tasks and (2) DF’s difference scores for both the slope and the PGA between the two grasping tasks. Together, these contrasts constitute tests for “strong/differential” or “classical” dissociations (Crawford et al., 2003; Crawford and Garthwaite, 2005). For all statistical tests, the alpha criterion for statistical significance was set to 0.05.

### Results

#### Peak Grip Aperture (PGA), Slopes, and the Peak Hand Velocity (PHV)

**Figure 2:**
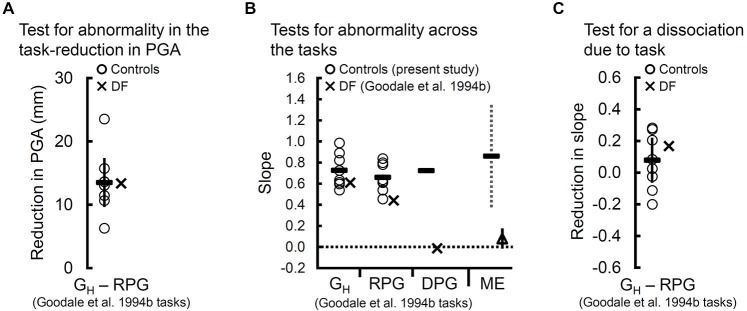
**Tests for dissociation using peak grip aperture (PGA) and the slopes of the controls (“O”s) and of DF (“X”s) across the natural grasps with haptic feedback (G_H_) and “real-time” pantomimed grasps (RPG) and tests for abnormality in DF’s slope across the G_H_ and RPG tasks. (A)** Reduction in PGA between the G_H_ and RPG tasks. The solid vertical bar reflects the 95% confidence interval and indicates a significant reduction in PGA moving from G_H_ to RPG for the controls. As can be seen, DF showed a similar reduction in her overall PGA. **(B)** Slopes relating PGA to target size for the controls (“O”s) and for DF (“X”s) for the G_H_ and RPG tasks. Dashes indicate the mean slope for the controls. DF’s slopes differ significantly from zero and are within the normal range in both tasks. For illustration, we included (1) the mean slope for the controls (solid dash) along with DF’s slope (“X”) computed from data reported by Goodale et al. (1994b) for the delayed-pantomimed grasping task (DPG); and (2) the mean slope relating grip aperture to Efron block width for DF (open triangle) and for the controls (solid dash) across 4 studies (Goodale et al., 1991; Westwood et al., 2002; Whitwell et al., 2014a, in press) of DF’s manual (perceptual) estimates (ME) of Efron block width. Evidently, the DPG task has a far more detrimental impact on DF’s slope than does the RPG task. In fact, DF’s slope in the DPG task failed to differ from zero (*p* = 0.9). Interestingly, DF’s particularly poor slope for the DPG task resembles those that are typically observed when she performs ME task. A 95% confidence interval around the controls’ mean ME slopes can be used to compare DF’s mean ME slope across those same four studies. Clearly, DF’s mean ME slope falls well outside the normal range. A 95% confidence interval to compare her mean ME slope against zero failed to yield a significant difference (*p* = 0.09). **(C)** The controls slopes for the G_H_ and RPG tasks do not differ significantly and, critically, the difference in DF’s slope between the two tasks falls within the range of differences observed in the controls. Thus, when compared to the G_H_ task, the RPG task affected DF’s slopes no differently than it did the controls.

The controls’ overall PGA was significantly larger when they performed natural grasps than when they performed RPGs, *t*_(7)_ = 8.23, *p* < 8 × 10^−5^ (see Figure 2A). A comparison of the difference in the overall PGA across the two tasks yielded no significant difference, *t*_(7)_ = −0.02, *p* = 0.98. In other words, the switch from natural grasps to RPGs affected DF’s overall PGA no differently than it did the controls’ overall PGA.

The controls’ slopes relating PGA to target size did not depend on whether they executed natural grasps or RPGs, *t*_(7)_ = 1.29, *p* = 0.24 (see Figure 2B). DF’s PGA was positively related to the size of the target in the natural grasping task (*t*_(28)_ = 6.01, *p* < 2 × 10^−6^) and in the RPG task, *t*_(28)_ = 2.98, *p* < 6 × 10^−3^. Importantly, DF’s slopes did not differ significantly from those of the controls when she performed natural grasps (*t*_(7)_ = −0.69, *p* = 0.61) or when she performed RPGs, *t*_(7)_ = −1.53, *p* = 0.17. Moreover, the test for dissociation yielded a null result, *t*_(7)_ = 0.5, *p* = 0.62 (see Figure 2C). In other words, DF’s slopes fell within the normal range regardless of whether she performed natural grasps or RPGs. Notably, DF’s slopes on the natural grasping task and the RPG task contrasts sharply with an absence of grip scaling on the DPG task in which her pantomimes were based on a memory of the previewed target (slope based on data reported in Goodale et al., 1994b) (*p* = 0.9; see Figure 2B).

The controls’ PHV was significantly slower when performing the RPGs than it was when they performed natural grasps, *t*_(7)_ = 2.79, *p* < 0.05.

**Figure 3:**
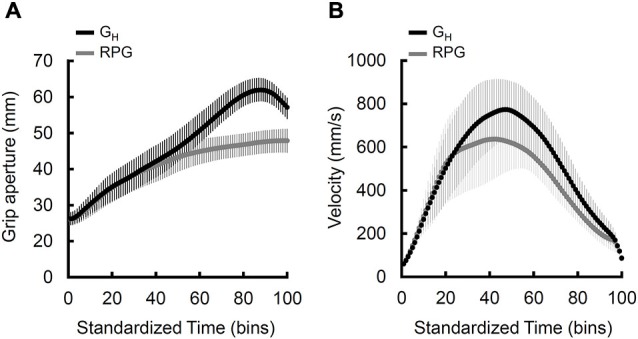
**Normalized grand mean grip aperture and velocity profiles for the natural grasps (black) and real-time pantomime grasps. (A)** Grip aperture normalized to 100 time bins for qualitative comparisons. Note that, overall, the real-time pantomime grasps lack a distinct peak and achieve lower grip aperture values than the natural grasps. The error bars reflect average within-participant standard deviations **(B)** Velocity of the wrist normalized to 100 time bins for qualitative comparisons. Note that, overall, the displaced-pantomime grasps appear to be executed more slowly than the natural grasps. The error bars reflect the between-participant standard deviations.

Finally, the time-normalized grip aperture (Figure 3A) and velocity (Figure 3B) profiles for the controls reveals a noticeable distinction between the natural and displaced-pantomime grasps that converges with the findings of Goodale et al. (1994b).

### Discussion

In this experiment we re-examined DF’s natural grasps and RPGs from an earlier study by Goodale et al. (1994b) by contrasting her performance on these two tasks with the performance of a new sample of normally-sighted control participants. When compared to natural grasps, the controls’ RPGs yielded smaller overall PGAs and slower PHVs. Thus, we replicated Goodale et al.’s findings but in a version of the task that the authors had modified before testing DF by reducing the number of possible target positions from three to one and increasing the number of targets from three to six. Although we were unable to examine DF’s PHV, we found that the RPG task reduced DF’s overall PGA just as much as it did for the controls. We also examined DF’s grip scaling in terms of the slope relating PGA to target size and for the controls. Somewhat surprisingly, we found that DF’s slopes fell within the control range in both tasks. Her intact performance on this task contrasts sharply with her performance on the DPG task in which (quite unlike controls) she shows no evidence of grip scaling at all. As we pointed out in the Introduction, one evident difference between the two tasks is the availability of real-time visual input about the target in the RPG task. In other words, in the RPG task, information about the target can be used in real time to program the movement parameters, including grip aperture. This is obviously not the case in the DPG task. Indeed, because the movement is being programmed in real time in the RPG task, the relatively intact visuomotor networks in DF’s dorsal stream could presumably mediate this programming. Although this line of argument is appealing, recent experiments suggest that real-time visual input is not sufficient for “normal” prehension (e.g., Bingham et al., 2007; Schenk, 2012a; Whitwell et al., 2014a,b).

Several years after Goodale et al.’s (1994b) investigation, Bingham et al. (2007) introduced a novel variant of a grasping task which was later adapted by Schenk (2012a) to re-test DF’s grasps. Noting that movements that lack feedback are often more variable, Bingham et al. (2007) hypothesized that goal-directed movements, such as grasping, are precise because they can make use of haptic feedback (what we are referring to here as haptics-based object information) for calibrating each movement. Thus, Bingham et al. reasoned, the slower pantomime grasping movements that Goodale et al. (1994b) observed could be due to a decrease in precision and the lack of haptics-based object information in the DPG and RPG tasks. Bingham et al. set out to test how the provision of periodic haptic feedback about the target object would affect the grasps of normally-sighted individuals. To do this, Bingham et al. used an ingenious mirror apparatus that allowed the participants to view a virtual target in the mirror. This way, the participants could be instructed to reach out behind the mirror towards the apparent position of the virtual target to grasp it. An identical copy of the virtual target could be positioned behind the mirror such that the virtual and hidden targets were spatially coincident. Critically, the arrangement allowed the experimenter the choice to deny the participants an opportunity to grasp a real cylinder by refraining from positioning one behind the mirror. In short, this setup allowed Bingham et al. to preserve both the real-time visual information about the targets and the target-directedness of natural grasps in these new grasping task variants. Similar to Goodale et al.’s findings, Bingham et al. found that when participants were consistently denied an object to grasp, they showed slower hand velocities, longer movement times, and lower overall PGA.

Several years following Bingham et al.’s (2007) study, Schenk (2012a) used a similar mirror-apparatus to re-examine patient DF’s grasps. He was motivated by the observation that the dissociation in grip scaling between DF’s grasping and her explicit perceptual estimates of target size might be due to the difference in the availability of haptic feedback about the target between grasping and perceptual estimation tasks. As Bingham et al. has suggested, haptic feedback might normally be used to calibrate actions. Perhaps DF has developed some abnormal reliance on this source of information that allows her to calibrate the programming of her grasps (see also Schenk, 2012b). Rather than providing DF haptic feedback for her perceptual estimations of target size, however, Schenk opted to divorce it from the grasping task as Bingham et al. (2007) had done. Critically, he found that DF’s grip scaling was abolished when haptic feedback was consistently denied and concluded that haptic feedback was required to calibrate DF’s grasping movements. Curiously, however, he did not appeal to the same pantomime-based explanation as Bingham et al. and Goodale et al. (1994b) had done in the past. Instead, he argued that DF uses haptic feedback to “compensate” for her visual perceptual deficit when reaching out to grasp objects (Schenk, 2012a). According to this line of reasoning, no distinction between visual processes for perception and those for skilled goal-directed action is required, because DF’s vision is merely degraded—haptics can help bootstrap her performance. As things turn out, this interpretation is incorrect, because DF’s inaccurate perceptual estimates of Efron width show no improvement when haptic feedback is available to putatively calibrate her estimates: she was permitted to reach out to pick up the Efron blocks immediately after each of her explicit estimates (Whitwell et al., 2014a, in press). Thus, DF’s dissociated performance on perceptual estimation and grasping tasks continues to support a fundamental distinction between dorsal and ventral stream object processing.

Nevertheless, one important factor was overlooked in both Schenk’s and Bingham et al.’s experiments: the participants in the “no haptic feedback” tasks of both studies were unlikely to have encountered anything other than “thin air” at the end of their reaching movements. For example, in Schenk’s study, the visual targets were vertically-standing cylinders 7 cm tall, requiring a horizontal grasping motion across the diameter of the visible target. In Bingham et al.’s investigation, the objects were shorter (though >3 cm in height), but the participants were explicitly instructed not to touch the surface of the table at the end of their reach and encouraged to adopt a particular approach that would minimize this possibility. At any rate, denying participants objects to grasp not only removed haptics-based object information in these studies but also any terminal tactile feedback about the end of the movement (Milner et al., 2012). This was not the case in Goodale et al.’s (1994b) study (and therefore in Experiment 1 of the present study) in which the participants, including DF, clearly made contact with the surface of the table next to the visible target. In fact, given DF’s normal grip scaling, the results from Experiment 1 support an important distinction between haptic-based object information and the information derived from terminal tactile input. Adapting the term as it was used by Bingham et al. and Schenk, we hereafter use “haptic feedback” in an overarching sense to refer to the denial of an object or even a proxy at the end of the movement (i.e., terminal tactile/haptic feedback).

Notably, a critical role for terminal tactile feedback in maintaining DF’s grip scaling is supported by the fact that DF scales her grip aperture to target size when she reaches out to “grasp” 2-D images of Efron blocks presented on a table top (Westwood et al., 2002). Furthermore, DF’s normal grip scaling in this 2D-grasping task cannot reasonably be attributed to the availability of online visual feedback to update her movements as they unfold or to update the programming of subsequent movements or even some sort of “visuo-manual matching” strategy, because she continues to show grip scaling to Efron width in the absence of any online visual feedback whatsoever (Whitwell et al., in press). Additional support for the role of terminal tactile feedback in maintaining DF’s grasps comes from the fact that her grip scaling is normal when she reaches out to grasp objects that vary in their visible (virtual) size but are always a constant, intermediate haptic size (Whitwell et al., 2014a, in press). In other words, haptics-based object information need not provide veridical information about the target width or edges of the visible goal object to maintain normal dorsal-stream mediated grasping. Indeed, the results of Experiment 1 indicate that DF shows normal grip scaling when terminal tactile feedback from the table surface is available to her, even when she performs RPGs. Interestingly, the results of Experiment 1 promote the real-time nature of a natural grasping task over the target-directedness of it *per se*. Thus, the two critical factors underlying DF’s grip scaling slope appear to be terminal tactile feedback and real-time visual input.

In the second experiment reported here, we addressed whether or not the task requiring DF and the control participants to reach out to a visible target that is not physically present results in grip scaling that resembles that of a more explicit pantomimed grasping task as Milner et al. (2012) suggest. After all, a desirable and novel feature of the grasping task used by Bingham et al. (2007) and Schenk (2012a) is that the resultant movements are programmed and executed in real-time and directed at the target—conditions under which the dorsal visuomotor system typically operates. Despite these similarities, there is some indication that the neurologically intact controls in Schenk’s (2012a) experiment showed an increase in grip-scaling and inter-subject variability (Whitwell and Buckingham, 2013). DF’s grip scaling to object size, as we pointed out earlier, was abolished in this task. Thus, the removal of haptic feedback appears to have changed DF’s grip scaling *and* that of the controls, but in different ways. Unfortunately, however, the controls’ grip-scaling with and without haptic feedback was never formally compared in that study. Thus, one aim of the second experiment reported here was to directly test whether removing haptic feedback from a target-directed grasping task influences grip scaling in neurologically-intact individuals. An additional aim (related to the first) was to directly contrast grasping in the target-directed task in which haptic feedback is removed against a variant of the RPG task in which the participants must imagine the visible target at a different location. This way, the responses when haptic feedback is denied in a target-directed grasping task could be compared to the responses in a task that quite obviously requires a pantomimed grasp. In order to implement these tasks, we adopted a mirror apparatus not unlike the one discussed above.

Finally, we took the opportunity that the mirror setup presented us to explore more systematically how the mirror itself might influence natural grasps. Although the mirror apparatus allows for the haptic and visual information about the target to differ, it has at least three possible drawbacks. (1) the mirror apparatus does not allow the participants to view their hand and limb throughout their grasping movement. The unavailability of any visual input about the hand and limb throughout the movement is of course quite different from what occurs with natural grasps. After all, normally when we reach out to pick things up, the hand and limb do not suddenly disappear from sight. A number of studies have shown that when vision is suppressed during the execution of a grasping movement in neurologically-intact individuals, grip aperture increases and, in many cases, the grip scaling slopes decrease (Fukui and Inui, 2006; Fukui et al., 2006; Whitwell et al., 2008, in press; Hesse and Franz, 2009, 2010; Whitwell and Goodale, 2009; Tang et al., 2014). In fact, DF shows similar changes in her grip aperture and grip scaling when vision is suppressed during the movement (Whitwell et al., in press). Presumably, these effects reflect an effort to ensure a sufficient margin of error in the absence of visual information that is normally used for online control. (2) When the participants make contact with the hidden object and pick it up, the virtual object remains stationary in the mirror. In short, there is a clear disconnect between what the participant sees in the mirror and what actually happens. (3) The mirror might be treated as an obstacle which has to be avoided. Any one or a combination of these three factors could have been responsible for reducing grip scaling in both normally-sighted individuals and in DF, because natural grasps that were directed at virtual targets in a mirror were contrasted against natural grasps that were directed at targets in plain view (Whitwell et al., 2014a). Thus, in an additional manipulation, we substituted a pane of glass in for the mirror to assess two effects of using a mirror: the removal of online visual input about the moving hand and limb, and the obvious disconnect between the behavior of viewed and hidden targets after contact. In total, therefore, we set out to test four tasks: grasping real targets (cylinders) viewed through a pane of glass (G_G–H_); grasping virtual targets viewed in a mirror with haptic feedback (G_M–H_); grasping virtual targets viewed in a mirror without any haptic feedback (i.e., no cylinder was present behind the mirror, G_M–NH_); and real-time pantomime grasps that were based on virtual targets viewed in a mirror but displaced to the side without any haptic feedback (RPG_NH_).

We grouped the task comparisons according to our apriori predictions: (1) that natural grasps directed at virtual targets (G_M–H_) would result in larger grip apertures than those directed at real targets viewed directly through glass (G_G–H_); and (2) in the absence of haptic feedback, target-directed grasping movements would resemble RPG_NH_ grasps that are directed towards an an imagined copy of the virtual target.

## Experiment 2

### Methods

#### Participants

Twenty-five self-reported right-hand dominant individuals (9 males) ranging from 17 to 33 years of age (*M* = 21.3, SD = 3.7), volunteered to take part in the second study. In a follow-up pair of control experiments that was prompted by some of our results, we tested an additional group of 18 self-reported right-hand dominant individuals (6 males) ranging from 18 to 32 years of age (*M* = 21.4, SD = 3.5). The participants in both groups provided written informed consent and were compensated $10 for their time. All experiments were approved by the local ethics committee and were in accordance with the Declaration of Helsinki.

#### Apparatus and Stimuli

**Figure 4:**
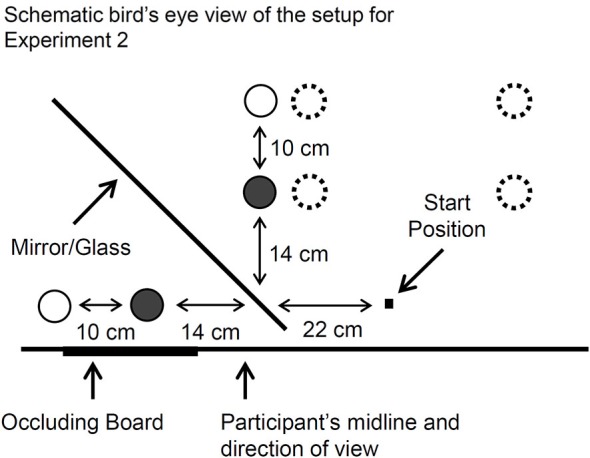
**A bird’s eye view of the mirror setup used for Experiment 2**. The cylinders (indicated by circles with a solid-line border) were placed in front of the mirror at one of two possible positions from trial to trial (the near position is indicated with a filled-in circle). The cylinder was hidden from direct view by an occluding board, and so the participants viewed a virtual cylinder in the mirror. An identical cylinder could be positioned behind the mirror (again indicated by circles with a solid-line border) such that it was spatially coincident with the apparent position of the virtual one. This way, haptic feedback about the object could be permitted (G_M–H_) or denied (G_M–NH_) by removing the cylinder from behind the mirror. In one of the tasks, the mirror was replaced with a pane of glass so that participants viewed the cylinder directly (G_G–H_). For the “real-time” pantomime grasping task (RPG_NH_), the participants imagined the virtual cylinder at the mirror-symmetrical position (dashed open circles) opposite a sagittal plane that was aligned with the start button. In a brief follow-up investigation, the RPG_NH_ task was modified such that the target was imagined immediately next to the visible one (also indicated with dashed open circles).

The apparatus and stimuli did not differ from that described in Experiment 1 except as noted below. The stimuli consisted of three pairs of black cylinders with diameters of 3.5 cm, 4.8 cm, and 6 cm and a height of 7 cm. Depending on the task, the workspace comprised either a mirror or a pane of glass positioned 45° from the edge of the table facing the participant. For all of the tasks that involved the mirror setup, the target cylinder was always positioned in front of the mirror. A vertically-standing occluding board was attached to the edge of the table that faced the participant. The occluding board was positioned to the left of the participants’ midline so as to block them from viewing the target cylinder directly. This way, the participant could only see the reflection of the cylinder (i.e., its virtual image) placed in front of the mirror. The occluding board was left in place throughout the experiment. The cylinders could be placed at two different positions in front of and (at corresponding positions) behind the mirror. The “near” target position was located 14 cm away from the mirror along the participant’s sagittal plane. The “far” position was located 10 cm farther away from the mirror along the same plane. The hand’s resting start position was a small black button located 22 cm to the right and 7 cm in front of the nearest target position (see Figure 4). Before the experiment began, the experimenter ensured that all of the participants were positioned close enough to the table so that they could grasp the objects at the farthest distance comfortably and without leaning forward. The experimenter also ensured that the participants could see each of the target cylinders binocularly in the mirror.

#### General Procedure and Design

Before each trial was initiated, the participants held the tips of their right index-finger and thumb together while depressing the start button. The participants were instructed to reach out, grasp, and lift up the cylinder using a precision grip (index-finger and thumb) as soon as the lenses of the goggles cleared. Participants were asked once they grasped and lifted the objects to simply move the objects to the center of the table. In all conditions, the lenses of the goggles remained transparent for 2.5 s following the participants’ release of the start button before returning to their translucent state (i.e., visual closed-loop feedback). Participants were asked to grasp the objects naturally, neither labored nor speeded. The experimenter explained the procedure for the upcoming task before each block of trials. The experiment was comprised of 4 blocks of 24 trials each for a total of 96 trials. Each block was dedicated to a different task. For each block of trials (i.e., for each task), the six combinations of target-cylinder size and location were presented 4 times each. The block order (i.e., task order) was counterbalanced across participants.

#### Grasping Real Targets Viewed Through a Pane of Glass

The participants viewed the cylinders through the pane of glass and were asked to reach out to pick them up as described in the previous section.

#### Grasping Virtual Targets Viewed in a Mirror With Haptic Feedback

The participants viewed the cylinders in the mirror. The experimenter ensured that the cylinder behind the mirror matched the one that the participants viewed. The participants were asked to reach out behind the mirror to pick up the cylinder as described in the previous section. Note that the mirror blocked the participants’ view of their hand during the movement. Thus, a comparison of this task with the one in which the participants grasped real targets viewed through a pane of glass tests the effect of online visual feedback of the hand and limb during the movement.

#### Grasping Virtual Targets Viewed in a Mirror Without Haptic Feedback

This task was identical to the task described in the previous section in all respects, except that, after the matched cylinder was placed behind the mirror, it was immediately removed and the trial then initiated. Positioning a target behind the mirror was done simply to preserve the overall “feel” and timing of the events between trials. Neither haptics-based object information nor any terminal tactile feedback was available in this task. In accordance with the instruction to simulate a real grasp, the participants were asked to refrain from sending their fingers or hand through the imagined cylinder.

#### Pantomime Grasping Visualized Copies of Virtual Targets Viewed in a Mirror

The participants viewed the cylinders in a mirror, but were asked to execute their grasps as if the cylinder was located to the right of where it appeared to be. This location was the right of the start button at a distance that equaled the distance from the visible cylinder to a sagittal plane aligned with the start button (see Figure 4). The experimenter explained this contingency to the participant and reinforced it by indicating the target locations for each of the two possible positions for the viewed cylinder. In accordance with the instruction to simulate a real grasp, the participants were asked to refrain from sending their fingers or hand through the imagined cylinder.

#### Data Processing and Statistical Analysis

The data were processed offline with custom software written in Matlab (Mathworks Inc., Natick, MA, USA). The positional data from the IREDs was low-pass filtered at 20 Hz using a 2nd order Butterworth digital filter. Grip aperture was computed as the Euclidean distance between the IRED placed on the thumb and the IRED placed on the index-finger, and the instantaneous velocities were computed for each of the three IREDs and for grip aperture.

The PGA was defined as the largest grip aperture within a search window that was designed to capture the forward-reach component of the movement. The beginning of this window, the movement onset, was operationally defined as the first of 20 consecutive sample frames (100 ms) in which the velocity of the IRED attached to the knuckle of the index-finger exceeded a threshold of 50 mm/s. The movement onset was also used to calculate the reaction time (RT). The end of the search window was defined as the first sample frame in which the velocity of the IRED fell below 150 mm/s. Linear regression of PGA on the widths of the cylinders was performed separately for each task and the resultant regression coefficient (slope, *b*_PGA_) relating the average increase in PGA (in mm) per incremental increase in cylinder width (also in mm) was computed. The PHV was defined as the peak velocity achieved by the knuckle IRED within the search window. One additional measure was operationally defined: the final grip aperture (FGA). The FGA was determined on the basis of grip stability (grip aperture velocity). Grip stability was used to identify the plateau phase of the grip aperture profile during which the participant holds the target (G_G–H_ and G_M–H_ tasks), pretends to hold a visible target (G_M–H_ task), or pretends to hold an imagined copy of a visible target (in the case of the RPG_NH_). Linear regression of FGA on the widths of the cylinders was performed separately for each task and the resultant regression coefficient (slope, *b*_FGA_) relating the average increase in FGA (in mm) per incremental increase in cylinder width (also in mm) was computed. Note that the *b*_FGA_ should be at or close to 1 for the natural grasps, and so the tests of this measure indicate how faithfully the participants reflected changes in target size from trial to trial in their FGA in the absence of haptic feedback.

To test for differences amongst the tasks, a one-way repeated measures Analysis of Variance (rmANOVA) was conducted separately for each of the dependent measures (RT, PHV, PGA, *b*_PGA_, FGA and *b*_FGA_) with Task as the main factor. The significant rmANOVAs were followed up with planned paired *t*-tests designed to test the specific effect of removing online visual feedback on the natural grasps and that of removing haptic feedback. The test of the former effect involved a comparison of the grasps directed at “real” cylinders viewed directly through a pane of glass (G_G–H_) and the grasps directed at “virtual” cylinders viewed in a mirror with haptic feedback (G_M–H_). The tests of the latter effect involved comparisons amongst the three tasks in which virtual cylinders were visible in the mirror: The G_M–H_ task, the variant without haptic feedback (G_M–NH_), and the real-time pantomime grasps directed away from the virtual cylinders and towards imaged ones without haptic feedback (RPG_M–NH_). With respect to this set of contrasts, it should be noted that the RPG_M–NH_ entailed online visual feedback. Therefore, we included a comparison of this task with the natural grasping task in which online visual feedback was available (i.e., RPG_M–NH_ vs. G_G–H_). Greenhouse-Giesser epsilon multipliers were applied to the degrees of freedom to all ANOVAs to compensate for potential violations of sphericity of the variance-covariance matrices. The F-statistics which were adjusted in this way are reported in-text as *F*_adj_. Violations of sphericity were assessed using Mauchley’s test and assessed at a liberal alpha criterion of 0.15 as Kirk (1995) recommends for tests of underlying assumptions. For all other statistical tests, the alpha criterion for statistical significance was set to 0.05.

### Results

#### Reaction Time

**Figure 5:**
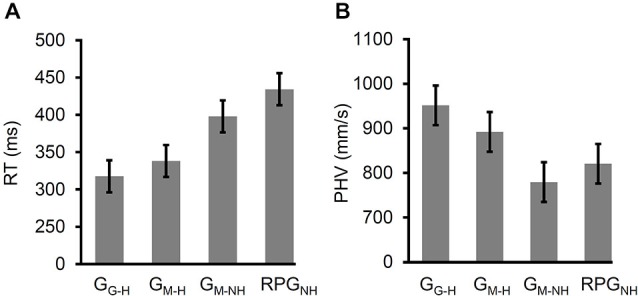
**Reaction time (RT) and peak hand velocity (PHV) across the four variants of the grasping task arranged (within each panel) from left to right as follows: grasps directed at real (i.e., viewed through a pane of glass) targets (G_G–H_) with haptic feedback, grasps directed at virtual targets (i.e., viewed in a mirror) with haptic feedback (G_M–H_), grasps directed at virtual targets with no haptic feedback (G_M–NH_), and the “real-time” pantomime grasps directed at imagined copies of the virtual targets (RPG_NH_)**. Note that the error bars reflect 95% confidence intervals extracted from the mean square error term from the rmANOVA (corrected for violations of sphericity where appropriate). **(A)** RT increased when a mirror was used rather than a pane of glass for target-directed grasps with haptic feedback. The RT increased further when haptic feedback was denied and increased further still when the participants performed displaced-pantomime grasps. **(B)** PHV slowed when a mirror was used rather than a pane of glass for target-directed grasps with haptic feedback. PHV slowed further when haptic feedback was denied and when the participants performed displaced-pantomime grasps.

The rmANOVA of the reaction times (RTs) yielded a significant main effect of Task, *F*_(3,72)_ = 26.7, *p* < 2 × 10^−11^, $\eta_{p}^{2}$ = 0.53 (see Figure 5A). There was no significant difference in the RTs between G_G–H_ and G_M–H_ (*t*_(24)_ = 1.75, *p* = 0.09), indicating no effect of online visual feedback on the velocity of the reach.

The RTs were slower for G_M–NH_ than the RTs for G_M–H_, *t*_(24)_ = 2.81, *p* < 0.01. In turn, the RTs for RPG_NH_ were significantly slower than those for G_M–H_, *t*_(24)_ = 6.52, *p* < 1 × 10^−6^. However, the RTs for RPG_NH_ were significantly faster than the RTs for G_M–NH_, *t*_(24)_ = 3.11, *p* < 5 × 10^−3^. Thus, the removal of haptic feedback induced a partial shift in the RTs towards pantomimed grasps. In other words, removing haptic feedback slowed the RTs and displacing the grasps slowed the RTs further still. Finally, the RTs for G_G–H_ were significantly faster than the RTs for RPG_NH_, *t*_(24)_ = 8.57, *p* < 1 × 10^−8^, suggesting that the slowing of RT that occurs when haptic feedback is denied occurs regardless of whether online visual feedback of the hand and limb is available or not.

#### Peak Hand Velocity (PHV)

The rmANOVA of PHV yielded a significant main effect of task, *F*_adj_(2,43) = 21.2, *p* < 1 × 10^−6^, $\eta_{p}^{2}$ = 0.47 (see Figure 5B). The PHV was significantly slower for G_M–H_ than for G_G–H_ (*t*_(24)_ = 5.34, *p* < 2 × 10^−5^), indicating a role for online visual feedback of the hand and limb in the velocity of the reach.

The PHV was significantly slower for G_M–NH_ than the PHV for G_M–H_, *t*_(24)_ = 5.87, *p* < 5 × 10^−6^. Furthermore, the PHV was significantly slower for RPG_NH_ than the PHV for G_M–H_, *t*_(24)_ = 2.29, *p* < 0.04. Finally, the PHV did not differ significantly between G_M–NH_ and RPG_NH_, *t*_(24)_ = 1.75, *p* = 0.09. Thus, the removal of haptic feedback resulted in a complete shift in the PHV towards pantomime grasps. In other words, regardless of whether the grasps were target-directed or not, the velocity of the reach was slower when haptic feedback was denied.

The PHV was significantly faster for G_G–H_ than it was for RPG_NH_ (*t*_(24)_ = 4.54, *p* < 2 × 10^−4^), suggesting that the slowing of PHV when haptic feedback is denied occurs regardless of whether online visual feedback of the hand and limb is available or not.

#### Peak Grip Aperture (PGA)

**Figure 6:**
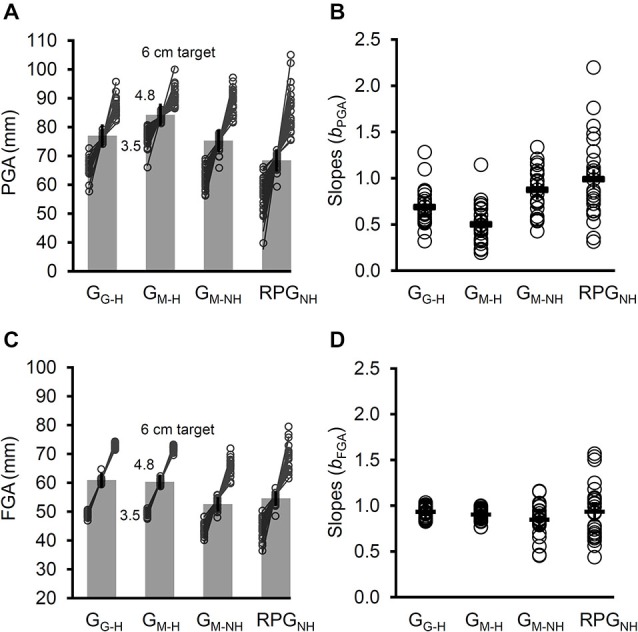
**The PGA, slopes relating PGA to target size (*b*_PGA_), the final grip aperture (FGA), and the slopes relating the FGA to the target size (*b*_FGA_) across the four tasks. (A)** The overall PGA increased when a mirror was used (G_M–H_) rather than a pane of glass (G_G–H_) for target-directed grasps with haptic feedback. For grasps directed a virtual targets, removing haptic feedback (G_M–NH_) reduced the PGA. The PGA was reduced further for the real-time pantomimed grasps (RPG_NH_). For each task the mean PGA for each target size is plotted for each participant. Evidently, denying haptic feedback increased the slopes. **(B)** The participants’ *b*_PGAs_ (open circles) and the mean *b*_PGA_ (dashes) for each task. The *b*_PGAs_ for G_M–H_ were smaller than those for G_G–H_, indicating a significant role for online visual feedback of the hand and limb. The *b*_PGAs_ were larger, however, whenever haptic feedback was denied, regardless of whether the grasps were target-directed (G_M–NH_) or not (RPG_NH_). **(C)** The overall FGA was reduced when haptic feedback was not available. Plotted for each task is the mean FGA for each target size for each participant. Even in the absence of haptic feedback, the FGAs were well-related to target size. **(D)** The *b*_FGA_ (open circles) did not differ amongst the four tasks. Note that the error bars reflect 95% confidence intervals extracted from the mean square error term from the rmANOVA (corrected for violations of sphericity where appropriate).

The rmANOVA of the mean PGA revealed a significant main effect of Task, *F*_adj_(2,47) = 18.5, *p* < 2 × 10^−6^, $\eta_{p}^{2}$ = 0.44 (see Figure 6A). The PGA for G_M–H_ was significantly larger than the PGA for G_G–H_ (*t*_(24)_ = 5.16, *p* < 3 × 10^−5^), indicating a significant effect of online visual feedback of the hand and limb in the offline and/or online updating of grip aperture in the natural grasping task.

The PGA was significantly smaller for G_M–NH_ than the PGA for G_M–H_, *t*_(24)_ = 3.4, *p* < 3 × 10^−3^. In turn, the PGA for RPG_NH_ was significantly smaller than the PGA for G_M–H_, *t*_(24)_ = 6.43, *p* < 2 × 10^−6^. However, the PGA for RPG_NH_ was significantly smaller than the PGA for G_M–NH_, *t*_(24)_ = 4.01, *p* < 6 × 10^−4^. Thus, removing haptic feedback induced a partial shift in the PGA towards pantomimed grasps. In other words, removing haptic feedback reduced the PGA, but displacing the grasp reduced the PGA further still.

The PGA for the G_G–H_ task was significantly larger than the PGA for the RPG_NH_ task (*t*_(24)_ = 4.36, *p* < 3 × 10^−4^), suggesting that the reduction in PGA when haptic feedback is denied also occurs regardless of whether online visual feedback of the hand and limb is available or not.

#### Regression Coefficients (Slopes) Relating PGA to Target Width

The rmANOVA performed on the slopes (*b*_PGA_) revealed a significant main effect of Task, *F*_adj_(2,52) = 24.4, *p* < 2 × 10^−8^, $\eta_{p}^{2}$ = 0.5 (see Figure 6B). The *b*_PGA_ for G_M–H_ was significantly smaller than the *b*_PGA_ for G_G–H_, *t*_(24)_ = 4.46, *p* < 2 × 10^−4^.

The *b*_PGA_ for G_M–NH_ was significantly larger than the *b*_PGA_ for G_M–H_, *t*_(24)_ = 7.31, *p* < 2 × 10^−7^. In turn, the* b*_PGA_ for RPG_NH_ was significantly larger than the* b*_PGA_ for G_M–H_, *t*_(24)_ = 6.33 *p* < 2 × 10^−6^. Finally, the *b*_PGA_ did not differ significantly between G_M–NH_ and RPG_NH_, *t*_(24)_ = 1.79, *p* = 0.09. Thus, the removal of haptic feedback resulted in a complete shift in the grip scaling slopes toward pantomime grasps. In other words, regardless of whether the grasps were target-directed or not, the slopes were larger when haptic feedback was denied.

The *b*_PGA_ for G_G–H_ was significantly smaller than the *b*_PGA_ for RPG_NH_ (*t*_(24)_ = 4.06, *p* < 5 × 10^−4^), suggesting that the increase in *b*_PGA_ when haptic feedback is denied also occurs regardless of whether online visual feedback of the hand and limb is available or not.

Finally, we opted to test for a difference in the *b*_PGA_ between the controls’ of Experiment 1 and the participants in the G_G–H_ task of Experiment 2 using an independent samples *t*-tests with appropriate adjustments for violations of homogeneity where necessary. We found no significant difference in the *b*_PGA_ across the two groups (*p* = 0.64), suggesting that the pane of glass did not affect the *b*_PGA_ in Experiment 2. Interestingly, pooling the no haptic feedback conditions in Experiment 2 (i.e., G_M–NH_ and RPG_NH_) to test for an effect of the absence of haptic feedback compared to terminal tactile feedback (i.e., the RPG task of Experiment 1) revealed an increase in the *b*_PGA_ for the former, *t*_(28)_ = 3.36, *p* < 3 × 10^−3^. Thus, the results of these additional tests support the findings of Experiment 1 that terminal tactile feedback helps “normalize” grip scaling slopes.

#### Final Grip Aperture (FGA)—Grip Stability at the End of the Reach

The rmANOVA of FGA revealed a significant main effect of Task, *F*_adj_(2,43) = 20.1, *p* < 2 × 10^−6^, $\eta_{p}^{2}$ = 0.46 (see Figure 6C). Not surprisingly, the FGA for G_M–H_ and G_G–H_ did not differ significantly (*t*_(24)_ = 1.41, *p* = 0.17.), presumably because this measure was constrained by the widths of the cylinders in these tasks. Thus, the removal of haptic feedback resulted in a complete shift in the FGA toward pantomime grasps. In other words, regardless of whether the grasps were target-directed or not, the FGA was smaller when haptic feedback was denied.

The FGA for G_M–NH_ was smaller than the FGA for G_M–H_, *t*_(24)_ = 5.3, *p* < 2 × 10^−5^. In turn, the FGA for RPG_NH_ was significantly smaller than the FGA for G_M–H_, *t*_(24)_ = 3.72, *p* < 2 × 10^−3^. However, the FGA for G_M–NH_ did not differ significantly from the FGA RPG_NH_, *t*_(24)_ = 1.69, *p* = 0.1. Thus, the removal of haptic feedback resulted in a complete shift in the FGA toward pantomime grasps. In other words, regardless of whether the grasps were target-directed or not, the FGA was smaller when haptic feedback was denied.

The FGA for G_G–H_ was significantly larger than the FGA for RPG_NH_ (*t*_(24)_ = 4.19, *p* < 4 × 10^−4^), suggesting that the reduction in FGA when haptic feedback is denied also occurs regardless of whether online visual feedback of the hand and limb is available or not.

#### Regression Coefficients (Slopes) Relating FGA to Target Width

The rmANOVA performed on the slopes relating FGA to target size (*b*_FGA_) indicated no significant main effect of Task, *F*_adj_(2,38) = 1.6, *p* = 0.22 (see Figure 6D), suggesting that, even in the absence of haptic feedback, participants on the whole took into account differences in the widths of the virtual cylinders when simulating their grip on them (in the case of G_M–NH_) or on imagined copies of the virtual cylinders (in the case of the RPG_NH_).

Finally, we examined the change in the slopes relating PGA to target size (*b*_PGA_) and those relating FGA to target size (*b*_FGA_) for each task (i.e., Δ*b* = *b*_FGA_ − *b*_PGA_). This analysis provides an indication of how consistent the slope was from the point in the response at which PGA was achieved (i.e., while the hand was in-flight) to the point at which the FGA occurred (i.e., while the fingers held the object tor simulated holding one). A significant Δ*b* was observed for G_G–H_ (*M* = 0.25, SD = 0.18, *t*_(24)_ = 6.88, *p* < 5 × 10^−7^) and G_M–H_, *M* = 0.4, SD = 0.21, *t*_(24)_ = 9.7, *p* < 9 × 10^−10^. In contrast, the Δ*b* for G_M–NH_ (*M* = −0.03, SD = 0.18, *t*_(24)_ = 0.76, *p* = 0.47) and RPG_NH_ (*M* = −0.06, SD = 0.28, *t*_(24)_ = 1.13, *p* = 0.27) failed to differ significantly from zero. Thus, the Δ*b* appeared to be largely driven by the availability of haptic feedback. To confirm this, a rmANOVA performed on the Δ*b* indicated a main effect of Task, *F*_(3,72)_ = 39.9, *p* < 3 × 10^−15^. Given the null findings amongst the tasks with respect to the *b*_FGA_, the differences in Δ*b* amongst the tasks are quite likely to have been driven by the differences in the *b*_PGA_ we reported above. Indeed, follow up tests (not reported) showed that this was true. Thus, the analysis of the Δ*b* indicates that in the absence of haptic feedback, the participants grip aperture faithfully reflected differences in the widths of the targets while their hand was in-flight and when it was simulating holding a visible or imagined cylinder.

#### Testing for Possible Methodological Issues With Respect to the Use of the Mirror

Given the significant differences between G_G–H_ (natural grasps directed at real targets viewed through a pane of glass) and G_M–H_ (natural grasps directed at virtual targets viewed in a mirror) tasks across a number of measures, we tested an additional group of participants (see Section Participants) to test for factors other than the online visual feedback of the hand and limb that could be driving this effect. In this follow-up experiment, we employed the G_G–H_ and G_M–H_ tasks (see Sections Participants, Apparatus and Stimuli, General Procedure and Design, Grasping Real Targets Viewed through a Pane of Glass, Grasping Virtual Targets Viewed in a Mirror with Haptic Feedback) however, the grasps in this additional experiment were performed entirely in open loop. In other words, the lenses of the goggles switched from a transparent state to a translucent one as soon as the participants’ fingers left the start button. Thus, the only difference between the tasks was that nature of the target image (one being virtual and the other real). If other methodological factors (e.g., subtle mismatch in the placement of the copy of the virtual target or differences in lighting) were responsible for the differences in grasping in the two tasks (rather than the differences in online sources of visual input) then we should replicate the pattern of results that we observed, because these differences would still be present despite the loss of online visual feedback throughout the movements.

**Figure 7:**
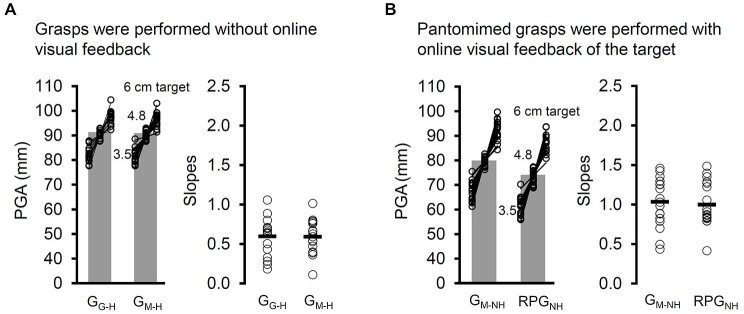
**(A)** The PGA and slopes (open circles, mean slope indicated by a dash) relating PGA to target width for the two variants of target-directed grasping tasks in which the participants executed their grasps in the absence of any online visual feedback (visual open loop). Grasps were directed at “real” (i.e., viewed through a pane of glass) targets (G_G–H_) and virtual (i.e., viewed in a mirror) targets (G_M–H_) in visual open loop (vision was occluded at the start of the movement). In the case of G_M–H_, haptic feedback was available when participants made contact with a spatially coincident duplicate that was positioned behind the mirror. Whether the grasps were directed at virtual targets or real ones made no difference across any of the dependent measures, including PGA and the slopes. **(B)** The PGA and slopes for the GDVT task in which haptic feedback was denied and the displaced-pantomime grasping (RPG_NH_) task in which the grasps were directed immediately to the right of the visible location of the target towards an imagined copy. Whether the grasps were directed towards or beside the virtual target did not affect the slopes, which appear to be quite steep in both tasks. Sending the hand to a location right beside the object did, however, reduce the overall PGA, just as it did for displaced-pantomime grasps to locations more distant from the location of the virtual target.

The results were clear: in the absence of any visual input throughout the grasping movements, viewing virtual or real targets did not significantly affect the RTs (*t*_(17)_ = 1.22, *p* = 0.24), PHVs (*t*_(17)_ = 1.16, *p* = 0.26), PGAs (*t*_(17)_ = 0.26, *p* = 0.8), or the *b*_PGA_, *t*_(17)_ = 0.14, *p* = 0.89 (see Figure 7A). Thus, the differences in the PHVs, PGAs, and *b*_PGA_ in Experiment 2 are unlikely to have been driven by methodological factors putatively introduced by using a mirror.

#### Removing Online Visual Feedback from the “Real-Time” Pantomime Grasping Task

As we have seen, the PGA for G_M–NH_ was smaller than the PGA for G_M–H_ yet larger than the PGA for the RPG_NH_. A similar result was observed for the RTs. Specifically, the RTs for G_M–NH_ were slower than those for G_M–H_ yet faster than those for RPG_NH_. The partial shifts in these measures for G_M–NH_ towards those observed in the pantomime grasping task (i.e., RPG_NH_) suggest that the target-directed nature of the G_M–NH_ task might have partially compensated for the effect of removing haptic feedback. It also possible, however, that the availability of online visual feedback of the hand and limb or the added shift in gaze or attention that the RPG_NH_ task demanded (as participants looked to towards the empty workspace to imagine a copy of the target) increased the RT. To test these possibilities, we carried out an additional experiment. We reasoned that altering RPG_NH_ so that the grasps were directed to a location immediately next to the virtual target should minimize differences between the two tasks in terms of the availability of visual feedback, shifts in attention, and other factors such as a difference in biomechanical constraint. A difference in PGA or RT following a comparison of the G_M–NH_ and modified RPG_NH_ (i.e., the grasps were directed to the side of the virtual target) would support the suggestion that the target-directed nature of the G_M–NH_ can at least partially compensate for an absence of haptic feedback.

Compared to G_M–NH_, the modified RPG_NH_ showed slower PHVs (*t*_(17)_ = 3.73, *p* < 2 × 10^−3^) (attributable to the modest overall reduction in distance the hand travelled in this task) and, importantly, a smaller PGA, *t*_(17)_ = 2.75, *p* < 0.02. Thus, directing the hand away from the target and towards an imagined copy appears to reduce the PGA no matter how far away from the visible object the hand is directed. The results also indicated no significant differences in the RT (*t*_(17)_ = 0.23, *p* = 0.82) or in the *b*_PGA_, *t*_(17)_ = 0.14, *p* = 0.89 (see Figure 7B) between G_M–NH_ and modified RPG_NH_.

#### Between-Groups Tests of the Regression Coefficients (Slopes) Relating PGA to Target Width

Testing the additional group of participants also afforded us an opportunity to test for a replication of one of the critical finding of Experiment 2 concerning the grip scaling slopes (*b*_PGA_). In a series of independent–samples *t*-tests involved the *b*_PGA_ of the G_M–NH_ and RPG_NH_ tasks from the first and second group of participants, and the G_M–H_ task from the first group, and in the series of independent–samples *t*-tests for the tasks in which the targets were virtual (i.e., viewed in a mirror) and the goggles remained clear for the duration of the movement (i.e., closed-loop with respect to the target). We adjusted the multiple *post hoc* independent–samples *t*-tests using Holm’s step-down Bonferroni procedure (Holm, 1979). The results, again, showed that the critical factor for this measure was the absence of haptic feedback. The *b*_PGA_ for G_M–NH_ (*p* < 7 × 10^−7^) and RPG_NH_ (*p* < 5 × 10^−8^) from the second group of participants were significantly steeper than the *b*_PGA_ for the G_M–H_ from the first group of participants. Furthermore, none of the tasks in which haptic feedback was denied differed between the two groups of participants (*p*_max_ = 0.14, uncorrected).

## Discussion and Conclusions

One of the principal aims of Experiment 2 was to determine whether or not removing both haptics-based object information and terminal tactile feedback (together referred to here as “haptic feedback”) from a target-directed grasping task shifts the response mode away from a natural one and towards a more pantomimed (i.e., simulated) kind as has been suggested by Milner et al. (2012). To do this, we compared target-directed grasps with (G_M–H_) and without haptic feedback (G_M–NH_) to pantomime grasps (RPG_NH_) in which the participants were asked to imagine a copy of the target in another location in the workspace and to grasp that imaginary object as if it were actually there (e.g., Goodale et al., 1994b; Holmes et al., 2013). We found that when participants reached out to grasp virtual targets, removing haptic feedback slowed RT and PHV, reduced PGA, increased the slopes relating PGA to the width of the target, and reduced the FGA. Just as important was the fact that the grasps directed at virtual targets (viewed in a mirror) without haptic feedback were statistically indistinguishable from the pantomime grasps in terms of the PHV, the slopes relating PGA to target size, the slopes relating FGA to target size, and the FGA, suggesting a complete shift across these measures towards pantomimed grasping following the removal of haptic feedback. The only measures that differed between the two “no haptic feedback” tasks were the RT and the magnitude of the PGA. It is important to acknowledge, however, that removing haptic feedback from grasps directed at virtual targets *slowed* the RTs and *reduced* the PGAs. In other words, both of these measures registered a shift in the direction *away* from natural grasps and *towards* the pantomimed ones.

An additional aim of Experiment 2 was to determine whether or not the mirror itself has an effect on the kinematics of target-directed grasps. After all, the mirror introduces three key differences when compared to natural grasps: First, the mirror blocks the participant’s view of their hand and limb as soon as the participant reaches behind it (removing re-afferent online visual feedback). Second once the participants make contact with the hidden object and the virtual target, the mirror imposes a disconnect between the felt movements of the hidden object and the apparently stationary target visible in the mirror. Although this effect might startle the participants at first, it is reasonable to suggest that the participants acclimate to this situation, growing more comfortable on subsequent trials. This says nothing, however, about any possible effects all of this might have on the unconscious “automatic” online control mechanisms that normally mediate grasping. Third, the mirror might act as an obstacle that the participants attempt to avoid. Given these considerations, we implemented an additional task in which the participants reached out to grasp target they viewed through a pane of glass. The pane of glass was the same size as the mirror and was positioned in the same way with respect to the participant. Compared to natural grasps directed behind the pane of glass, the ones directed behind the mirror resulted in slower PHVs, larger PGAs, and shallower slopes. Nevertheless, it was possible that some other aspects of the mirror task may have played a role. We ruled these factors out in a control experiment in which we removed online visual feedback altogether for both tasks. In this control experiment, all the differences between the grasps directed behind the mirror and the grasps directed behind the pane of glass completely disappeared, strongly implicating a role for one or more of the sources of online visual feedback outlined above in the programming and updating of target-directed grasps. Given Connolly and Goodale’s (1999) null findings concerning the magnitude of the PGA and the fact that the participants in that study were permitted a view of the target and the hand making contact with the target, then the results of the current investigation suggest a significant role for vision during the contact and manipulation phase of the grasping movement in the programming of grip aperture on subsequent grasps.

Many of the additional findings in the present investigation can be explained through the changes in task demands and differences in the availability of visual and haptic input. For example, the overall reduction in PGA and FGA in the absence of haptic feedback (see also Bingham et al., 2007; Fukui and Inui, 2013) is likely due to the removal of the physical constraints that the object imposes on the fingers and, therefore, the minimum magnitude that the grip aperture would normally be required to achieve a suitable grasp. Without the physical constraints imposed on the fingers and hand by an actual object, there would be (1) no consequences for consistently under-sizing grip aperture, such as knocking the object away; and (2) less effort (and perhaps even more comfort) in opening the hand a smaller amount. The FGA, being a measure of grip stability when the target is being held, would necessarily be restricted by the sizes of the cylinders. We speculate that the selective removal of haptics-based object information might also lift this restriction and result in a similar reduction in FGA. Nevertheless, unlike the FGA, the PGA was affected by both haptic feedback and online visual feedback of the hand and limb. Specifically, providing online visual feedback and removing haptic feedback each effected reductions in the PGA. The effect of online visual feedback of the hand and limb on PGA observed in the present study is in line with previous findings following a comparable manipulation (Whitwell et al., 2014a,b) and is also in line with the broader literature on the effects of removing online visual feedback entirely (e.g., Jakobson and Goodale, 1991; Whitwell et al., 2008; Whitwell and Goodale, 2009; Hesse and Franz, 2010). The effect of removing haptic feedback on PGA observed in the present study is also in line with previous reports in which terminal tactile feedback was almost certainly denied (Bingham et al., 2007; Fukui and Inui, 2013). Interestingly, (although not always explicitly tested), a similar effect on PGA appears to occur in the absence of haptics-based object information when short (e.g., ~1 cm in height) block-like stimuli (or even 2D images) are used in which the fingers are highly unlikely to avoid touching the surface of the table at the end of the reach (e.g., Westwood et al., 2002; Cavina-Pratesi et al., 2011; Holmes et al., 2013). If we assume an additive model of the effects of online visual feedback and haptic feedback, then consideration of the details of the tasks of the present study readily explain the findings involving FGA and PGA.

In keeping with an appeal to differences in task demands, we should point out that we required the participants to refrain from sending their hand and fingers through the visible or imagined target for the tasks in which haptic feedback was removed. We would argue that most (if not all) tasks in which the participants *simulate* grasps carry with them analogous instructions, regardless of whether such instructions are stated explicitly by the experimenter or are tacitly understood by the participant. Critical to this is (1) any consideration the participants might give to the sizes and positions of the target in a situation in which the target is not actually there; and (2) how well the participants understand what their hand does when they reach out to pick up a goal object. It seems likely that these factors account for the increase in RT when haptic feedback was denied. A similar appeal to differences in task demands can explain the additional increase in RT that occurred when the grasps were directed at an imagined copy of the visible object. Unlike the other grasping tasks, the instructions for the pantomime grasps required the participants to imagine a copy of the visible target at a different location. Presumably, participants would first look at the visible target and then look towards the location where they were to imagine a copy of that object before or shortly after they initiated their response. In contrast, in the target-directed grasping tasks (with and without haptic feedback), the target’s viewed position and the location to which the participants sent their hand are one and the same. We suspect that the addition of a preparatory shift in gaze in the pantomime grasping task likely increased the RT relative to the target-directed grasping task in which haptic feedback was denied. It is possible that the biomechanical difference in the direction that the participants sent their hand and limb in pantomime grasping task or the availability of online visual feedback might also play a role in the increase in RT. We should point out, however, that in the control experiment in which haptic feedback was denied, RT did not depend on whether the participants directed their hand towards the virtual target or beside it. In other words, the difference in RT between pantomimed and target-directed grasps without haptic feedback was abolished when the pantomime task was modified to minimize differences in shifts in gaze or attention, biomechanical constraints, and online visual feedback. Furthermore, we note that online visual feedback did not influence the RT of natural grasps in the current study—a finding consistent with previous investigations of natural grasps with and without online vision (e.g., Whitwell et al., 2008; Hesse and Franz, 2010). Thus, it seems unlikely that this factor can account for differences in RT in the absence of haptic feedback.

In contrast to the RTs and the PGA, the PHV, the slopes relating PGA to target size, the slopes relating FGA to target size, and the FGA were not affected by the added requirements of pantomime grasps when compared to the target-directed grasps without haptic feedback. In other words, for these measures of movement execution, the target-directedness of the response was not a critical factor. Instead, the removal of haptic feedback about the object appeared to dominate, independent of whether the grasp was directed to a visible or an imagined target. In line with Bingham et al.’s (2007) finding, without haptic feedback the PHVs were slower. The participants likely approached the targets more cautiously and deliberately, presumably because they were simulating what they would do if an object was actually there, making sure that their fingers did not go through the visible or imagined object. Importantly, the slopes increased relative to the slopes for grasps that received haptic feedback, approaching a 1:1 relationship between changes in the width of the target and changes in PGA. In fact, the slopes in these tasks resemble those observed during manual estimation tasks in which the participants indicate the width of a visible object by opening their thumb and index-finger a matching amount (e.g., Daprati and Gentilucci, 1997; Haffenden and Goodale, 1998; Pettypiece et al., 2010; Schenk, 2012a; Whitwell et al., 2014a, in press). Thus, the increase in the grip scaling slope when haptic feedback is not available would appear to reflect the deliberate consideration given to the sizes of the targets in these simulated grasps. As Whitwell and Buckingham (2013) noted, removing haptic feedback from a real-time grasping task appears to increase the grip-scaling slopes (Schenk, 2012a). In our experiment (see also Byrne et al., 2013) we explicitly tested this and found that, in the absence of haptic feedback, the slopes do, in fact, increase relative to natural grasping tasks. Interestingly, on a task that is not unlike the delayed-pantomimed grasping task devised by Goodale et al. (1994b), the slopes appear to increase relative to those observed on a natural grasping task regardless of whether vision of the workspace is available at the time of the movement or not (see Fukui and Inui, 2013). Overall, it seems reasonable to conclude that in the absence of haptic feedback, the geometric properties of the target are taken into explicit consideration when planning and programming the grasp. Thus, DF’s poor grip scaling slope when haptic feedback is consistently denied (Schenk, 2012a) can be attributed to a switch in the kind of response she provided towards a more pantomimed or simulated one as Milner et al. (2012) suggested. Interestingly, as we showed in Experiment 1, the provision of some proxy *next* to the visible target (in our case the surface of the table) has a normalizing influence on DF’s and the controls’ slopes. This finding adds to a growing body of work indicating that DF’s slope remains normal provided that real-time visual input is available along with tactile feedback from a proxy of the target (Westwood et al., 2002; Whitwell et al., 2014a,b).

Additional support for a distinction between haptics-based object information from a real (3D) object and tactile feedback from a proxy comes from studies of the influence of a mismatch between the haptic and visual size of target objects. When normally-sighted participants reach out to grasp objects in which the apparent visual width of the objects differs from their felt width, they typically show some adaptation in their PGA to the actual (i.e., the felt) size of the target—even though they continue to scale their grip aperture to the visual width of the target (e.g., Gentilucci et al., 1995; Säfström and Edin, 2004, 2008; Pettypiece et al., 2010). In fact, DF responds in an identical manner, suggesting that (1) the ventral stream is not required for the updating of grip aperture to reflect the real size of a target and that (2) veridical haptics-based object information is not required for DF to maintain normal grip scaling to trial-to-trial changes in the visual sizes of targets (Whitwell et al., 2014a,b). Rather, DF’s dorsal stream can exploit terminal tactile feedback to update her grip aperture on subsequent grasping movements and to maintain normal visuomotor processing of target shape to program movements parameters like grip aperture. Thus, it seems reasonable to conclude that (1) provided real-time visual input is available, tactile feedback from the surface of the table is sufficient to keep the visuomotor networks in DF’s dorsal stream engaged; and that (2) the damaged areas of DF’s ventral stream are not necessary for grip scaling for grasps that are directed towards the table surface next to a visible object.

Since Goodale et al.’s (1994a) study, pantomime grasps have been used in many kinematic investigations and is considered a tool to test the role that perception plays in the visual control of skilled actions. For example, the PGAs of pantomime grasps have been shown to be more susceptible to the Muller-Lyer illusion than natural grasps (Westwood et al., 2000). In addition, the within-subject variability of the PGAs of pantomimed grasps, but not natural ones, obeys Weber’s Law (i.e., the variability of the PGA increases linearly with target width; Holmes et al., 2013; although see Foster and Franz, 2013). In fact, even the movement preparatory time for pantomimed grasps, but not for natural gasps, is increased by the holistic object-perception that is thought to underlie Garner interference (Ganel and Goodale, 2003, 2014). Moreover, patient IG, who suffers from optic ataxia following damage to her PPC shows a paradoxical improvement in the correlation between her PGA and target width when she executes pantomime grasps following a delay period compared to natural grasps (Milner et al., 2001). Finally, provided the object is visible, the hand kinematics of magicians (who routinely pantomime actions to deceive their audiences) look far more like those of natural grasps than they do those of non-magicians (Cavina-Pratesi et al., 2011). In all of these studies, haptic feedback about the object was denied but not terminal tactile feedback about the end of the movement. Thus, tactile feedback from the tabletop is not enough to preserve *all* of the kinematics of a real grasping movement. Indeed, when neurologically-intact individuals pretend to pick up 2D images, the variability of their grip aperture scales with target size as Weber’s law would predict (Holmes and Heath, 2013), just as it does for pantomimed grasps (Holmes et al., 2013). Furthermore, grasps that are directed towards 2D objects invoke holistic processing (Freud and Ganel, 2015) in which the irrelevant and relevant target dimensions interact to influence processing times. This is not so for grasps that are directed at 3D objects (e.g., Janczyk and Kunde, 2012; Eloka et al., 2014; Freud and Ganel, 2015).

Importantly, it remains to be seen whether the cognitive or perceptual effects associated with pantomimed grasps are indeed mediated by ventral stream processing as is commonly assumed. An interesting future direction might be to test DF’s pantomime grasps for evidence of holistic processing (e.g., Garner interference) and relative sensitivity to stimulus magnitude (e.g., Weber’s law). Interestingly, pantomime grasps directed to the workspace next to a visible object fail to elicit preferential activity in the temporal-occipital areas in healthy participants, uniquely recruiting, instead, regions in the supramarginal gyrus, middle intraparietal sulcus, and supplementary motor area of the right hemisphere (Króliczak et al., 2008)—areas that remain intact in DF. These findings, combined with those of Goodale et al. (1994b) and the present study, suggest that a delayed pantomime grasping task would invoke preferential activity in areas of the occipito-temporal cortex of healthy individuals that are damaged in DF. Interestingly, these areas are in fact recruited when reach-to-grasp movements are based on a memory of the target, albeit in the context of a delayed grasp (as opposed to a delayed pantomime grasp) which received haptic feedback about the remembered object at the end of the reach (Singhal et al., 2013). Thus, although pantomime grasps with tactile feedback invoke cognitive and perceptual influences that are absent in natural grasps, some of these influences (e.g., the effects of holistic processing on movement preparation time, or of stimulus magnitude) might well emerge from a combination of visual processes in the ventral stream and the inferior parietal cortex of the right hemisphere.

In summary, the current study shows clear evidence that the removal of haptic feedback induces a shift from natural towards pantomimed (simulated) grasps, as suggested by Milner et al. (2012). The pattern of changes in the grasps kinematics, longer initiation times, slower movements, and steeper slopes were indicative of a more deliberate process of responding in which the participants explicitly took into account the metrics of the object, the location to which they were directing their hand, and the path that their hand and fingers would take. Furthermore, as Fukui and Inui (2013) have pointed out, the reduction in grip aperture that followed the removal of haptic feedback presumably reflects a natural consequence of the removal of a physical object, which, normally, would impose a constraint on the grip aperture of a natural grasp. Thus, the removal of haptic feedback also changes the task incentives. Without haptic feedback, there is no obvious consequence for an inaccurate grasp. These results and those of other investigations highlight the importance of haptics-based object information, or, at the least, terminal tactile feedback, in maintaining normal grasps which, we have shown here with patient DF, depend on intact dorsal pathways.

## Conflict of Interest Statement

The authors declare that the research was conducted in the absence of any commercial or financial relationships that could be construed as a potential conflict of interest.
